# Application of mesenchymal stem cells in bone regenerative procedures in oral implantology. A literature review

**DOI:** 10.4317/jced.51186

**Published:** 2014-02-01

**Authors:** Jose A. Viña, Marya El-Alami, Juan Gambini, Consuelo Borras, Jose Viña, María A. Peñarrocha

**Affiliations:** 1 Master of Oral Surgery and Implantology. Valencia University Medical and Dental School. Valencia, Spain; 2 Department of physiology. Valencia University Medical School. Valencia, Spain; 3 Assistant professor of Oral Surgery. University Medical and Dental School. Professor of Master of Oral Surgery and Implantology. Valencia University Medical and Dental School. Valencia, Spain

## Abstract

Objective: The aim of this work was to review de literature about the role of mesenchymal stem cells in bone regenerative procedures in oral implantology, specifically, in the time require to promote bone regeneration. Study Design: A bibliographic search was carried out in PUBMED with a combination of different key words. Animal and human studies that assessed histomorphometrically the influence of mesenchymal stem cells on bone regeneration procedures in oral implantology surgeries were examined. Reults: - Alveolar regeneration: Different controlled histomorphometric animal studies showed that bone regeneration is faster using stem cells seeded in scaffolds than using scaffolds or platelet rich plasma alone. Human studies revealed that stem cells increase bone regeneration. - Maxillary sinus lift: Controlled studies in animals and in humans showed higher bone regeneration applying stem cells compared with controls. - Periimplantary bone regeneration and alveolar distraction: Studies in animals showed higher regeneration when stem cells are used. In humans, no evidence of applying mesenchymal stem cells in these regeneration procedures was found. Conclusion: Stem cells may promote bone regeneration and be useful in bone regenerative procedures in oral implantology, but no firm conclusions can be drawn from the rather limited clinical studies so far performed.

** Key words:**Mesenchymal stem cells, bone regeneration, dental implants, oral surgery, tissue engineering.

## Introduction

Embryonic stem cells are derived from blastocysts (1) and are considered as pluripotent cells as they are able to form all the body´s lineages (endoderm, mesoderm and ectoderm) (2). Mesenchymal stem cells (MSC) are one type of adult stem cells, that are able to give rise to tissues of mesodermal origin such as dentine, bone, or peri-odontal ligament (3). Bone regenerative potential of MSC has been evaluated in bone defects in animals (4-6). The histomorfometric results of these studies showed higher bone regeneration using MSC seeded on a scaffold that with the scaffold (4-6) and similar results than with autogenous bone grafts (6).

Surgical bone regeneration procedures such as guided bone regeneration or maxillary sinus augmentation, are well established (7), but methods that do not require the collection of autogenous bone, but that ensure sufficient bone formation in a short period of time are interesting (8). To achieve this goal several agents like bone morfogenertic proteins (9,10), platelet rich plasma (PRP) (11), melatonin (12) or stem cells (13) have been studied with different outcomes. Using tissue engineering, different recent studies have applied MSC in bone regeneration in oral implantology. MSC have been used in alveolar regeneration (14), maxillary sinus augmentation (15), periimplantary bone regeneration (16) or alveolar distraction (17), in human and animal studies. Results of these histomorphometric animal studies (14,15) have shown that MSC enhance bone regeneration in a shorter period of time than biomaterials alone. Clinical human studies have also demonstrated higher bone regeneration when MSC have been applied (13,18).

The aim of this work was to review de literature about the role on MSC in bone regenerative procedures in oral implantology, specifically, in the time require to promote bone regeneration.

## Material and Methods

A bibliographic search in PUBMED with a combination of different key words: mesenchymal stem cells, bone regeneration, dental implants, tissue engineering, dental pulp stem cells, periodontal ligament stem cells, apical papilla stem cells, deciduous teeth stem cells, oral implantology, oral surgery. Moreover, the references of the articles were also assessed. English articles published until 2011 were evaluated. Animal and human studies that assessed the influence of MSC on bone regeneration procedures in oral implantology surgeries were examined. Studies about alveolar bone regeneration, maxillary sinus lift, periimplantary bone regeneration and alveolar distraction were identified.

-Inclusion criteria

Due to the number of studies performed no strict inclusion criteria could be performed. Only studies that used MSC to performed bone regeneration where histologic or histomorfometric analysis was performed were included. The studies should specify the moment where the analysis was carried out.

•Clinical Human studies: Clinical trials, case-controls and case series were included. Case reports were excluded.

• Animal studies: Controlled studies were included. Non-controlled studies were excluded.

When we applied these inclusion criteria, 26 articles were specifically analyzed.  Table 1 shows the included articles.

**Table 1:**
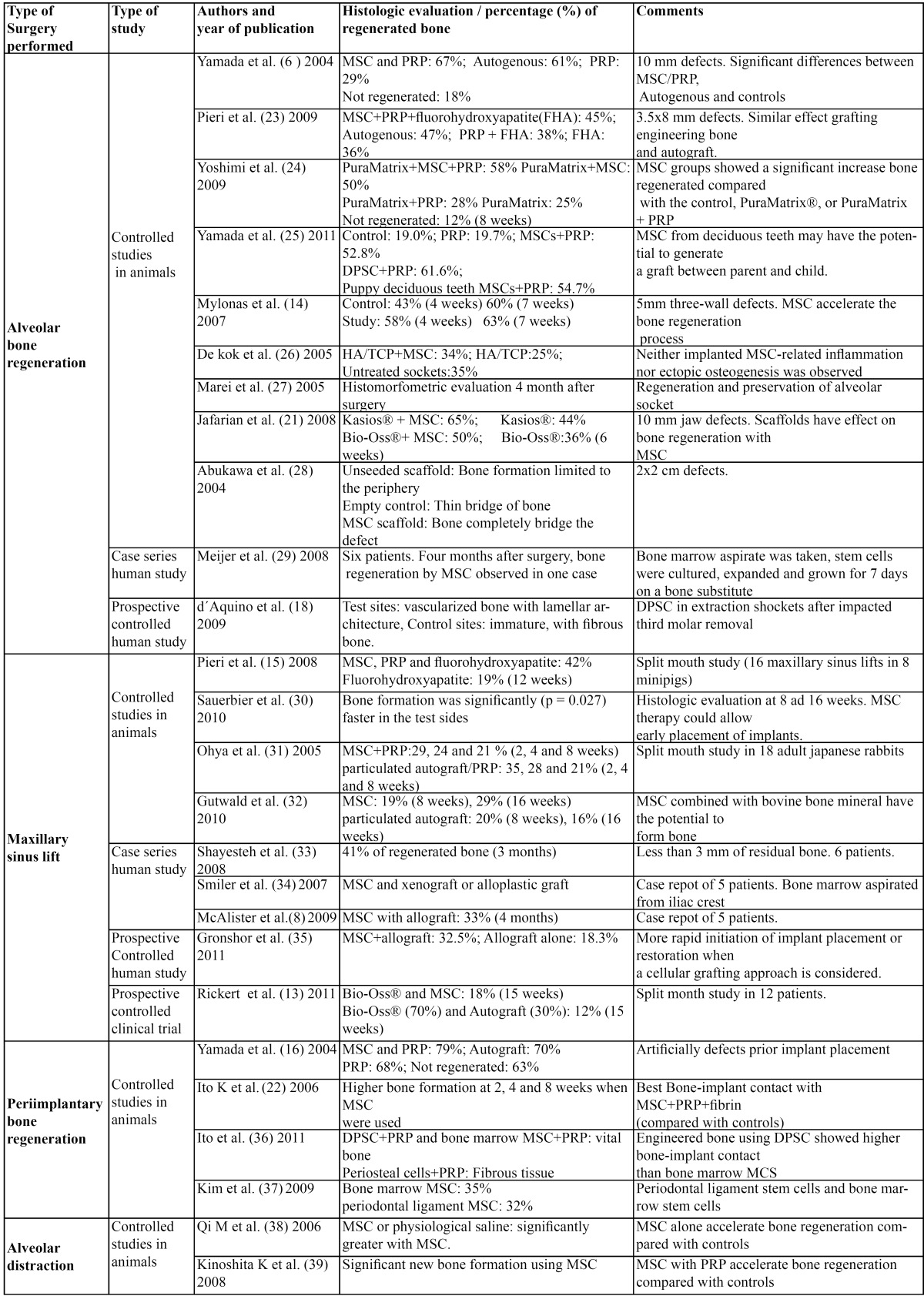
A summary of the results of the included articles about application of MSC for bone regeneration in implant dentistry.

Data about study design, bone regeneration surgery performed and time of histologic -histomorfometric study was carefully taken in account. Different MSC sources, scaffolds and growth factors were used in the reviewed articles so no differences between studies could be performed in this sense.

## Mesenchymal stem cells in bone regeneration procedures applied to oral implantology

Tissue engineering is an emerging interdisciplinary field, which applies principles of life sciences and engineering towards the development of biological substitutes that restore, maintain and improve the function of damaged and/or lost tissues (19). In tissue engineering, scaffolds are required to give support to cells or other structures. Scaffolds are made of biodegradable material, which is a biocompatible product that is gradually reabsorbed once implanted in the body, usually due to enzymatic degradation (20). The following sections review the most relevant animal and human studies, in which stem cells are used in the field of bone regeneration. All the studies mentioned apply the principles of tissue engineering stem cells. Thus scaffolds like xenografts (21), hydroxyapatite (23) or fibrin (22) are used. In different studies (6,16), PRP was used as a scaffold for the stem cells, but also as a graft material to evaluate its regeneration potential.

-Alveolar bone regeneration

•Animal studies

Different studies have compared bone regeneration using MSC and PRP. Yamada et al. (8) in 10 mm defects, 8 weeks after grafting, showed that MSC and PRP were as effective as particulate autograft (67% and 61% of bone regenerated respectively); PRP alone caused a 29% of bone formation and 18% was found in non-regenerated cases. Using edentulous ridge in minipigs, Pieri et al. (23) observed higher bone formation with autogenous bone (47%) or MSC, PRP and fluorohydroxyapatite (45%) than with PRP and fluorohydroxyapatite (38%). Yoshimi et al. (24) observed similar effects using dogs. Moreover, Yamada et al. (25) evaluated the bone regeneration in mandibular defects with bone marrow MSC, dental pulp stem cells (DPSC), and stem cells from deciduous teeth from their puppies; they observed bone formation with the 3 groups compared with the controls (empty or PRP). They concluded that MSC from deciduous teeth may have the potential to generate a graft between parent and child.

Mylonas et al. (14) applied MSC to 5mm three-wall defects and performed histomorphometric analysis in two different moments. Four weeks after grafting, bone regeneration was higher in MSC group (58%) than in controls (43%). After 3 weeks more, histomorphometric results were similar in both groups (63% and 60% respectively). These results showed that, although bone regeneration occurs when the necessary conditions were present, MSC accelerate the process.

Other studies exposed that MSC can also accelerate bone formation in alveolar shockets (26,27), in 10 mm jaw defects (21), or even in greater defects (2x2 cm) (28).

Clinical human studies

Meijer et al. (29) implanted MSC to regenerate alveolar bone defects in 6 patients. Four months later, bone regeneration was observed in 3 of them. The authors reported that only in one case, regeneration was performed more than 7 mm separated from the preexisting bone walls, and for the authors, only in this case, regeneration was due to MSC. Of the 11 implants placed in 5 patients one failed. d´Aquino et al. (18) performed a clinical trial to assess the bone regeneration of DPSC in postextraction, impacted third molar sockets. They grafted one socket with DPSC in a collagen sponge scaffold and compared with non-grafted control. X-ray analyses and probing depth, showed better results in the study group. Three months after surgery, histologic analysis showed well vascularized bone with lamellar architecture in the study sites, while control sites were immature, with fibrous bone.

-Maxillary sinus lift 

•Animal studies

Controlled studies in animals using MSC to increase bone regeneration in maxillary sinus lifts have been performed. Pieri et al. (15) in a split mouth study, performed 16 maxillary sinus lifts in 8 minipigs. MSC, PRP and fluorohydroxyapatite were placed in one side, and fluorohydroxyapatite alone in the other. Twelve weeks later, they observed 42% of regenerated bone when MSC were grafted and 19% in controls. Sauebier et al. (30) in a study in sheep showed that MSC accelerate bone regeneration compared with a xenograft alone. Other studies (31,32), observed 8 weeks after the intervention, similar bone regeneration whether grafting with MSC construct or with particulated autograft.

•Clinical human studies

Clinical case series studies using MSC with different biomaterials have been performed. Shayesteh et al. (33) performed a clinical study in 6 patients, where MSC and beta-tricalcium phosphate/hydroxyapatite were used as graft material in maxillary sinus lifts with less than 3 mm of residual bone. Three months later, histomorfometric study showed 41% of regenerated bone. Thirty implants were placed in the grafted areas of which two failed. Other authors (34), performed maxillary sinus lift and onlay grafts using MSC mixed with xenograft or allograft in 5 patients. They observed bone regeneration in the grafted zones by histological and histomorfometric analysis. McAlister et al. (8) performed direct sinus lifts to 5 patients using MSC with an allograft and they saw an average vital bone of 33% at 4 months. No implant survival was recorded.

Gronshor et al. (35) in a controlled study, compared the regenerated bone grafting with allograft and MSC or allograft alone; they observed 3,7 month after, a 32.5% of vital bone in the cellular scaffold group compared with the 18.3% in the control.

Very recently the first controlled clinical trial using engineered bone stem cells in maxillary sinus elevation has been performed. Rickert et al. (13) executed a split mouth study in 12 patients where they randomly grafted one maxillary sinus with xenograft and bone marrow MSC, and the other with xenograft (70%) and autograft (30%). Fifteen weeks later, the histomorfometric study showed significantly higher bone formation in the MSC group (18%) than in the control (12%).

-Periimplantary bone regeneration

•Animal studies

Animal studies have evaluated MSC combined with PRP in order to enhance periimplantary bone regeneration. Yamada et al. (16) regenerated artificially created osseous dehiscence with MSC and PRP, PRP alone, autograft, or non- regenerated controls. Two month later, implants were place in the grafted areas, and after another 2 month, a histometrically study was performed. Results showed that in those cases regenerated with MSC and PRP or with autografts, bone height remained constant. However, in those regenerated with PRP alone or not regenerated (controls), there was exposure of the implant threads. Ito et al. (22) performed a similar study in which implants were placed in bone with defects regenerated with the following graft materials: MSC with PRP and fibrin, MSC with fibrin, fibrin or non regenerated. The best periimplantary bone regeneration and highest bone-implant contact occurred when MSC with PRP and fibrin was used.

Different studies have evaluated the regeneration potential of stem cells harvested from different sources. Ito et al. (36) compared osseointegration of dental implants and tissue-engineered bone using DPSC, MSC from bone marrow or periosteal cells. Mandibular defects were filled with cells from one of these three sources and PRP. Eight weeks later, implants were placed, and after another 8 weeks, the percentage of bone-implant contact was assessed. Results sowed 67%, 62% and 39 % of bone-implant contact for the DPSC, bone marrow MSC and periosteal cells respectively. In the stem cells groups vital bone was observed while in the periosteal cell and the empty control fibrous tissue was found. Kim et al. (37) observed higher periimplantary bone regeneration graf-ting the defects with bone marrow MSC than with periodontal ligament MSC (35 and 32% of regenerated bone respectively).

-Alveolar distraction

•Animal studies

Qi et al. (38) evaluated the effect of MSC on bone formation in 40 male rats; after distraction was completed, the gap was filled with MSC or physiological saline. Callus formation was found in both treatment groups, but they observed, on days 27 and 55 after the onset of the distraction, higher radiodensity and bone formation when MSC were used. Kinoshita et al. (39) studied the effect of MSC and PRP on bone formation in alveolar distraction in 12 rabbits. A radiologic and morphometric study was performed 2, 3 and 4 weeks post-distraction. Better results were obtained when the gap was infiltrated with MSC and PRP than when physiological saline or PRP alone were used.

 Table 1 shows the results of the included studies.

## Conclusions

Controlled animal studies reveal that stem cells combined with PRP accelerate bone regeneration in oral implantology surgeries. This combination has shown higher capacity than PRP alone to promote bone regeneration. Controlled, long-term follow up clinical human studies have not been performed, but so far, no serious complications related to stem cells grafting (like systemic inflammatory response) have been reported. There is only one very recently clinical trial performing maxillary sinus lift using stem cells seeded on a xenograft showing similar morphometric results than autografts with xenograft. The interesting results of this clinical work as well as those of previous animal studies, indicate that stem cells may promote bone regeneration and be useful in human oral implantology. Many different scaffolds are used; for this reason and the heterogeneity between studies, no big conclusions can be performed yet. More clinical controlled studies applying stem cells are necessary to validate in humans, the favorable results observed in animals.
